# Breaking good? Young people's mechanisms of resilience, resistance and control

**DOI:** 10.1111/1468-4446.13164

**Published:** 2024-11-21

**Authors:** Claire Fox, Jo Deakin

**Affiliations:** ^1^ University of Manchester Manchester UK

**Keywords:** inequalities, interventions, marginalisation, resilience, young people

## Abstract

The conventional understanding of resilience often portrays it as a positive outcome emerging from adverse situations. This perspective frequently shapes interventions aimed at bolstering resilience among individuals considered to be in need. Drawing upon data from a European study, this paper contends that young people's apparent ‘latent rejection’ of favourable opportunities, or their deliberate choice to remain in precarious situations despite having some agency, should be recontextualised as unconventional but valid expressions of resilience. Instead of framing resilience solely as an aspirational concept, we propose a reframing that emphasises its role in coping with and surviving challenging circumstances. Furthermore, we advocate for the adoption of Mason's ‘safe‐uncertainty’ model to foster a more practical form of resilience. This approach towards a more sustainable resilience could be valuable in other fields dealing with those populations labelled as ‘vulnerable’, ‘problematic’ or ‘disadvantaged’, and it can, we argue, enhance decision‐making skills, and promote the development of robust support networks.

## 1 INTRODUCTION

The concept of resilience has had something of an identity crisis, its meaning often taken for granted and associated assumptions diluted within everyday discourse. Such presumptions often preclude a more critical exploration of the concept; specifically, who is setting the ‘gold standard’ of resilient behaviour that so many are measured against, and why. Research critically examining resilience and how ‘risky youth’ access and use social resources related to the concept is relatively limited (Lenkens et al., 2020). Resilience has frequently been conflated with desistance^1^ (Fitzpatrick, 2011; Lenkens et al., 2019)—the name given to the process of abstaining from criminal or antisocial behaviour (Maruna, 2001), usually after a period of frequently engaging in offending activities and replaced by a more prosocial identity—and used as a byword for ‘breaking good’. This has been evident in two related senses; firstly, resilience is an element that contributes to or promotes desistance. Secondly, resilience and desistance are two concepts that, whilst they can be conceived of as separate entities, have much in common, not least the perceived need to utilise protective factors in order to move towards positive outcomes (Lenkens et al., 2020; Ungar, 2011), avoiding relationships labelled as unhealthy or risky (Weaver, 2016; Zolkoski & Bullock, 2012). By taking (culturally) normative characterisations of behaviours, ability, competency, attitude and so on, as the gold standard, definitions that feature in related policy and practice fail to appreciate unconventional forms of resilience (Walsh, 2002). The ‘socially constructed and sanctioned’ expectations of resilient behaviour are set as the norm (Hutcheon & Lashewicz, 2015, p. 43; Hutcheon & Lashewicz, 2014; Walsh, 2002). In the UK, these discourses have been increasingly visible in policies aimed at young people (Bull & Allen, 2018). This has implications for those who may be caught up (at risk of becoming so) in the criminal justice system. These ‘targets of resilience’ (Gill & Orgad, 2018, p. 479) are urged to ‘break good’; that is, to demonstrate conventionally resilient behaviour, despite the context within which they live. Those who are seen as lacking positive (so‐called) resilient character traits are judged as failing (Mu, 2020) or problematic, and may face further sanction.

The appeal of the notion of resilience is undeniable, not least for those making or enacting policy. In our rapidly changing world, young people encounter a multitude of challenges, from economic uncertainties to social and technological disruptions, all of which require demonstrations of resilience. It is unsurprising that resilience has emerged as a powerful analytical framework. Whilst there have been various moves to offer a comprehensive—or at least sufficient—definition, universally agreed‐upon understanding remains elusive. Nevertheless, there has been a notable shift away from the persistent assumption that resilience solely implies positive adaptations or behaviours in response to traumatic or challenging circumstances. In adding to this important body of work, we delve into case study data from four European countries, analysing the narratives of resilience among young individuals facing multiple social and structural disadvantages. Our primary arguments revolve around two key points. Firstly, we offer further support for the redefining of resilient behaviour to encompass a broader range of behaviours, including those that deviate from the expected norms.

Secondly, drawing on Mason's (1993) work on risk, change, and positionality, we contend that for resilience to thrive amidst the uncertainties, disadvantages, and challenges faced by young people, it is imperative to promote initiatives and practices that encourage a state of ‘safe‐uncertainty’. This entails enabling individuals to reflect on their position concerning any present or looming risks, empowering them to contemplate and make changes in their lives. Consequently, our findings hold significant implications for shaping policies and practices in the domains of youth work and youth justice and beyond, particularly within the context of addressing inequalities.

While this paper focuses on young people facing multiple disadvantages, the reconceptualisation of resilience proposed here, and the related application of safe‐uncertainty have potential implications far beyond this specific population and context. By expanding our understanding of what constitutes resilient behaviour, this framework could be applied to various fields such as mental health, education, organisational psychology, and disaster response. It offers a more inclusive lens through which to view human responses to adversity across different life stages and sociocultural contexts.

### 1.1 Understanding and reclaiming resilience

Understandings of resilience are commonly associated with progressive outcomes, such as demonstrating ‘strength and growth’ (Green et al., 2021, p. 864) or the ‘bounce back factor’ (Walklate et al., 2012, p. 189). In a definition that neatly summarises how resilience is viewed in much of the literature, Masten and Powell (2003 cited in Ungar, 2008, p. 220) consider resilience as ‘patterns of positive adaptation in the context of significant risk or adversity’. This perception of resilience as a ‘heroic’ conception (Estêvão et al., 2017, p. 9) has been commonly seen across work on the topic (cf. Brooks, 2006; Gilligan, 2000; Schoon, 2006; Zolkoski & Bullock, 2012). In their review of resilience research, Aburn et al. (2016, p. 12) found the notion of triumphing over adversity writ large within this literature, often accompanied by words such as ‘flourishing’, ‘thriving’ and ‘succeeding’. Resilience has frequently been viewed as being ‘all about changing in order not to be changed’ (Walker, 2020, p. 1).

Resilience is widely accepted as a process, rather than a static trait, with an expectation that levels of resilience can grow but also diminish over time or in response to different stimuli. There has been a preoccupation with identifying risk and protective factors that enable or prohibit resilient behaviour(cf. Masten, 1994).^2^ For others, using an ecological lens to understand resilience is essential, not only to appreciate how interactions with the social world influence the ability to cope with times of stress but also to be sensitive to the notion that those who do not develop sufficient resilience—those who supposedly ‘give in’—are susceptible to being labelled as ‘non‐resilient’ or ‘failing’, which for some can be equated to victim‐blaming (Brooks, 2006).

There remain deficits and gaps that have troubled studies of resilience, which in turn have provided a site of criticism and confusion (Fergus & Zimmerman, 2005; Luthar et al., 2000). Much of the literature locates the concept firmly with the actor, neatly sidestepping the need to consider the structural context within which they are located (Harris et al., 2017; Mohaupt, 2009), which, therefore, remains unchallenged (Gill & Orgad, 2018; Pelling, 2010).

However, the preoccupations with risk/protective factors have begun to give way to more nuanced conceptions of resilience, challenging the notions of successful outcomes that work on resilience had been so wedded to (Hutcheon & Lashewicz, 2014). These approaches promote the view that resilience cannot be considered as something that is exclusively rooted in the individual nor does it view the individual as a passive bundle subject to various influences within the wider social and political context. It is instead to be seen as a ‘constellation of factors that interact’ (Ungar, 2006, p. 58), where there exists ‘chaotic, complex, relative, and contextual’ relationships between the various factors (Ungar, 2004, p. 342). Understood in this way, resilience represents a set of (socially) constructed negotiations and relationships that are used to navigate a given situation.

Moving away from the individualistic approach to resilience allows for a critical lens to be cast over the motivations for promoting such a view. In short, it allows us to ask who is defining resilience and to what end:

Put bluntly, there is nothing inherently negative or positive about resilience, as it is entirely contingent on who is wielding it, and for what political purposes.

(DeVerteuil & Golubchikov, 2016, p. 144)

It is not a neutral, politically‐disconnected concept. Instead, it has come to prominently feature in many aspects of everyday life, particularly across governmental realms of health, education, social welfare, and employment but also on social media, ‘lifestyle’ magazines and websites, and reality television (Allen & Bull, 2018; Burman, 2018; Gill & Orgad, 2018). Whilst the meaning of resilience has all too often been assumed, Evans and Reid (2013, p. 93) argue that ‘[b]eneath this veneer of common sense, however, lurks a dark and dehumanising political agenda’.

The definitional difficulties with the concepts have not stemmed increasing numbers of public policies and reports that seemingly ‘aim to develop ready‐made, off‐the‐shelf toolkits for resilience‐building’ (Davoudi, 2012, p. 229). Resilience has been constructed and used within policy and political circles as a means for pushing responsibilities for wellbeing, coping with adversity and overcoming traumatic events back into the remit of the individual and the community, in a manner that suggests something of a ‘do it yourself’ approach to developing and maintaining resilience (Walklate et al., 2012). These forms of individualisation and responsibilisation of the consequences of adversity become particularly pertinent for young people facing existing social inequalities.

For those who wish to use it in this way, its hazy and flexible definition can be advantageous, with resilience acting as:

a conveniently nebulous concept incorporating shifting notions of risk and responsibility bounded within a reconstituted governance framework—all of which can engender confidence and potentially facilitate the transfer of costs away from the state to the private sector and communities.

(White & O’Hare, 2014, p. 947)

Resilience is constructed as an individualised responsibility, allowing governments and their agencies to shrug off the responsibility of so‐called ‘needy’ people (Andres & Round, 2015; DeVerteuil & Golubchikov, 2016). Such an approach can blame individuals and communities for their own adversity, rather than critically examining the unequal distribution of resources, power, and opportunities that contribute to social inequalities (Bradbury‐Jones et al., 2017; Joseph, 2013). It is no accident that resilience is oft‐regarded as an individualistic trait, in a way that ‘chime[s] with the neoliberal enterprise of self‐responsibilisation and free[s] the system from accountability’ (Mu, 2021, p. 826). Despite impressions to the contrary, resilience is arguably a ‘tool of governance’ (DeVerteuil & Golubchikov, 2016, p. 144) and the enthusiastic embracing of resilience within a range of policy documents is far from coincidental (Joseph, 2013). This politicised ‘rise of resilience thinking’ is seen to ‘resonate[s] with the general promotion of responsibilisation, self‐organisation, and adaptation, particularly with regard to self‐reliance under neo‐liberalism’ (McKeown et al., 2021, p. 114; see also Joseph, 2013). As recent analysis has shown, the increased political use of resilience is:

intimately related to the cutting back, closure, and privatisation of public services, working as part of an individualising and blaming strategy in which people are made responsible for their own well‐being, always already at risk of being recast as ‘failing’ or ‘non‐resilient’.

(Gill & Orgad, 2018, p. 479)

Put simply, the social framing of resilience should not be ignored. Giving the concept of resilience, a mask of depoliticisation effectively neuters criticism of state responsibility or complicity in sustaining structural inequalities (cf. Boyden & Cooper, 2007; Gill & Orgad, 2018). The assumed neutrality of resilience allows it to be framed as the adaptability of individuals and not of the system (Joseph, 2013). The consequences for this go well beyond the context of this paper and the effects are wide‐ranging. This is compounded by the ‘resilience creep’ (Walklate et al., 2012, p. 186), which has seen it used widely within policy and practice literature. As the language of resilience becomes ubiquitous, wheedling itself into ‘the nooks and crannies of everyday life’ (Littler, 2017 cited in Gill & Orgad, 2018, p. 478), its presence becomes accepted and unquestioned.

Given the definitional tangles that plague understandings of resilience, it is unsurprising that cultural specificities have been largely neglected (Ungar, 2006, 2008). The definitions are frequently rooted in a Western‐centric position, and it has become a highly gendered and classed concept (Gill & Orgad, 2018). Resilience conceptualisations often fail to account for the differing social and cultural contexts (Bottrell, 2009; Ungar, 2013) nor the intersecting identities that shape experiences of adversity and resilience (Crenshaw, 1989).^3^

A more critical examination of resilience within social contexts is necessary to avoid co‐opting assumptions of normative behaviour as benchmarks of youth resilience (Bottrell, 2009; Ungar, 2004). This article, therefore, employs a framework that understands resilience as something that:

cannot be dis‐embedded from these contested and multiple processes—it cannot be thought of as a ‘thing’ or ‘outcome’, but rather always arises from the situated practices, politics and meanings of negotiation in specific sites.

(Harris et al., 2017, p. 198)

We argue that there needs to be a move towards encouraging safe‐uncertainty (Mason, 1993) so that interventions for young people at risk of offending or who have an offending history are effective. Through this, professionals working with young people can support them in developing resilience, which in turn can better aid reflection and confidence in making positive changes. We conclude by adding that this is no easy feat given the heightened sensitivities of ‘risk’ within social life, particularly in a youth justice system that continues to fall back on risk‐based practices that pre‐date its overall commitment to a *child first* ethos (Bateman, 2020). And so, whilst Mason's ‘safe‐uncertainty’ allows for flexibility and the acknowledgement of unpredictability, this will be counter to many institutional systems that tend to avoid uncertainty, instead relying on rigid, risk‐averse approaches that make it difficult to implement.

### 1.2 Methodology

This paper draws on findings from four case studies conducted as part of the PROMOTING YOUTH INVOLVEMENT AND SOCIAL ENGAGEMENT (PROMISE) project^4^ in the United Kingdom (UK), Portugal, Estonia and Spain that focused on young people who were or had been in conflict with social control figures or sites, such as those related to the criminal justice system, the education system and state‐run care settings.^5^

Within each of the case studies, there were multiple sites of data collection (see Table 1). These sites encompassed a range of statutory and third‐sector interventions, such as youth clubs, support groups and mandatory criminal justice programmes, that focused on supporting those young people who had been deemed as being ‘at risk’ of (re)offending or were viewed as ‘troubled’. Contact with participants was made through these interventions. They were primarily aged between 15 and 24, with a small number of outliers ‐ younger participants involved in interventions and older participants reflecting on their experiences.

**TABLE 1:** Case studies: Participants and data collection.

Country^8^	Research sites	Number of interviews	Identifying gender of interview participants	Ages of participants
Estonia	Mandatory community‐based probation intervention including post‐custodial intervention and alternative to custody	24	21 male	15–27
			3 female	
Portugal	Mandatory non‐custodial youth justice intervention	26	17 male	15–24
	Two mandatory second chance education schools for young people excluded from mainstream schooling		9 female	
Spain	Four voluntary support groups for young people described as NEET (typically without work permits or in other vulnerable social situations)	21	15 male	15–32
			6 female	
UK	Mandatory community‐based youth justice intervention for young people undergoing ISSP.	21	11 male	13–30
	Three voluntary youth clubs targeting young people ‘facing multiple disadvantages’		10 female	
	Voluntary support group for young people leaving the care system			
Total	A mix of mandatory and voluntary interventions designed to support and/or manage young people	92	64 male	Majority between 15 and 24
			28 female	

The qualitative fieldwork comprised of three stages: observations, interviews and where possible, arts‐based methods. A total of 92 interviews took place across the 4 case studies.^6^ Interviews were semi‐structured and designed to elicit narratives from the participants, prioritising their frame of reference. The interviews were adapted to be of relevance to the setting in which the participants belonged and to ensure it was meaningful. In addition, focus groups occurred in Estonia (three participants) and the UK (six and eight participants in two separate groups). Observations helped to inform our knowledge of the various settings, as well as build familiarity and trust between the researchers and the young people. The research also incorporated arts‐based data collection, primarily by way of photo elicitation and accompanying group sessions to discuss the emerging themes these produced. The aim of this tripartite of methods was to promote a collaborative and participatory approach to data collection and analysis.

The data collected was analysed using a meta‐ethnographic synthesis method (Pilkington, 2018), which provided ‘a means for synthesising the findings of large, multilingual qualitative data sets in order to see the bigger picture, while minimising the loss of contextual differentiation’ (Deakin, Fox, & Matos, 2022, p. 660). The data went through a dual analysis process: each case study was first reviewed and coded by at least 2 researchers to promote reflexive discussion of the cases, before being translated into English where necessary. A skeleton coding system allowed ‘for variation in cases while retaining an overall structure necessary for the second stage’ (Deakin, Fox, & Matos, 2022, p. 660). This second stage involved recoding the data in accordance with the foundational and emergent themes of the research. To achieve rigour and consistency across the coding process, a team of researchers from the wider multinational research group reviewed the key themes and messages from the data. The research went through a thorough ethical review process, with informed consent being confirmed at every stage of the fieldwork process, and the anonymisation of any identifying elements of the data, including the artistic outputs and interviews, and use of pseudonyms in research materials.

Despite such rigours, limitations and gaps in the research remained. We were particularly mindful to side‐step the pitfalls of imposing our research agenda and methods onto the participants in a top‐down fashion. The perils of this are two‐fold; the research will fail to pick up on the things that matter to the young people by dictating the research agenda, and secondly, the research will unintentionally (re)stigmatise the young people involved or reinforce existing stigma (for full discussion see Deakin, Fox, & Matos, 2022). To mitigate against these challenges, the research was guided by local‐level steering groups, which included young people and professionals working with young people and incorporated a methodological design that aimed to prioritise the views and beliefs of the participants, whilst also being respectful of their opinions. Through the arts‐based methods, in particular, participant engagement was prioritised.

### 1.3 The realities of resilience: From ‘latent rejection’ to ‘generative action’

Many of the young participants faced multiple layers of disadvantage. For example, Helen's background was characterised by family addiction, parental indifference and engagements with the criminal justice system:

So, my mum was an alcoholic, so she drank very heavily; and she was, dare I say, bat‐shit crazy…. I was supposed to go back [home] to my mum, and she was supposed to pick me up from social services, and she didn't turn up

(Helen, aged 30, UK)

Experiences of victimisation or social harm were not uncommon amongst the participants, although there was a noticeable avoidance of labelling, by themselves or others, as ‘victims’.

Their ability to cope with conflicts with authority figures and other challenging circumstances in their lives, including the various forms of stigma they encountered (Deakin, Fox, & Matos, 2022), was evident through a range of behaviours and actions that do not neatly fit with the pro‐social, neoliberal notions of resilience. The various individualised strategies that the young people used when responding to conflict were apparent across the four case studies. For many, the reflexive ability to carve out geographical, psychological and/or social space from conflict and any associated exacerbators offered a way to manage these conflicts, avoid new ones and/or minimise any negative repercussions. These responses may appear to be unorthodox or nonsensical to the outside observer. However, taken from the perspective of the young person, it offered a way of exercising their agency within a life that may be bound by various constraints, affording little room for them to flex their ability to control a given situation. For Liam, an acute sense of limited agency was palpable in his discussions:

And they expect me to go up there for ten to just do some shit. And I’m thinking, ‘Are you tapped in the head? I wanna go back home.’ But I’m thinking, ‘But I can’t go back home. If I go home, I get kicked off this course, then [further sanctions].’

(Liam, aged 15, UK)

For the Promoting Youth Involvement and Social Engagement (PROMISE) project, the notion of agency was not confined to pro‐social or proactive actions; across the case studies, behaviours which may be considered negative or apathetic were also captured and recast as operations of agency. In some cases, exercising what (limited) agency was available could be seen as a way of developing and maintaining resilience. For example, in a bid to avoid conflict with teaching staff and to distance himself from the criminal behaviour of his peers, Liam decided to stop attending his mainstream school, and then his first pupil referral unit:

I was like, “You know what, fuck you, I’m not coming into your school.” ….So I stopped going in and then the next thing is like…. “Right, you’re being moved” […] Gone to it…. And I’m thinking, ‘For fucks sake … and then half the time you just don’t wanna go in because it’s either the teachers just fucking waffle on shit or students just say, “Oh, yeah, I did this last night and it was sick, and we robbed a car.”

(Liam, 15, UK)

For Liam, who was living in a care home and serving a community sentence, this offered a way to reduce the potential of further criminal involvement and remove himself from situations that threatened conflict with teachers, something he was mindful could ignite further trouble. According to Ungar (2011), such behaviours, while not conventionally viewed as adaptive, represent resourceful in their own way ‐ responses to systemic barriers, enabling individuals to maintain a sense of control and agency in their lives.

Samantha had a difficult upbringing involving neglect from both parents. When expecting her child, there was little support on offer from the social care sector, who she found to be ‘judging’ her based solely upon her own parents' failures. Samantha felt a lack of control over the events during the pregnancy and afterwards, and her efforts to engage with social services were unsuccessful:

When I talk to them, they just ignore you. When you try to have a conversation with them, they just blank you out.

(Samantha, 24, UK)

Samantha's way of coping with social workers doing ‘nothing’ was to remove herself from the situation ‐ she would ‘just get angry and then walk out’.

These strategies of resilience were often obscured or normalised by the participants but demonstrated that forms of ‘hidden resilience’ (Ungar, 2006, p. 53), whilst not readily detectable, are still instances of coping with or surviving challenging circumstances, nonetheless.

Our data offered various examples of collective and individual activities to promote change. The findings also suggested that other, less normative responses should be recognised and reframed as acts of engagement and resilience, despite being negatively viewed. Rather than seeing anti‐social behaviour, apathy and resistance to interventions as a failure in resilience, these actions and behaviours can be better understood as some of the ways resilience manifests itself within that young person's life. In keeping with some of the more recent resilience literature, we suggest that by reframing what we understand by expressions of resilience to include the full spectrum of action, reaction, and inaction, we can more fully understand young people's responses as a corpus of coping mechanisms.

Resilience can surface through resistance to the wont of authority figures, including parents, teachers, Youth Justice team members, and social care professionals. This resistance, sometimes repeated over days, weeks or beyond, can be seen as a form of coping with the world. Although it may not initially be recognised as such, repeated ‘bad behaviours’ can be a form of social action that some of the young people in the PROMISE project were found to engage in. Moreover, ‘doing nothing’ (Corrigan, 1993, p. 103) or ‘latent rejection’ (Deakin, Fox, & Matos, 2022, p. 15) could be perceived as a form of self‐preservation, primarily enabling the young person to continue in their status quo, even when that includes maintaining ‘risky behaviour’ and/or remaining in conflict with authorities. Resistance, including these forms of ‘expressive activities’ (Mythen, 2012, p. 402), can be seen as another mode of resilient behaviour. Engaging in interventions, therefore, can represent a step into the unknown, taking them outside of the familiar/comfortable, and encouraging changes in behaviour.

For many of the young people who participated in the PROMISE project, being resilient may mean something less ambitious, such as arriving at a mandatory intervention only marginally late or even simply getting out of bed at all. It may be that they did not provoke further arguments with the police, which could have led to more serious repercussions, suggesting that they are learning to navigate, perhaps in less obvious ways, their day‐to‐day lives in ways that minimise the potential for conflict (cf. [Fox et al., 2022 and Mythen's (2012: 403) discussion of ‘acts of circumnavigation’). For example, Brian felt that because he had a reputation for ‘being naughty’, he was treated more harshly and unfairly than his peers. He cited incidents where teaching staff would ‘abuse [their] authority’ to physically prevent Brian from leaving a class. This, for Brian, was wrong because ‘he's a teacher, not […] a security guard’ but when these instances occurred, Brian decided that he would need to ‘accept’ this behaviour, as a trade‐off for maintaining his reputation, thus demonstrating his own individualised form of resilience:

Because like I knew that with a reputation like that, that people are gonna be on my case. I seen it growing up, like I seen it before. So, like I accepted it at the time. For me, it's just like a big game, innit.

(Brian, 18, UK)

Accepting the social constructionist view of resilience, and the presence of socially desirable normative goals and behaviour, Kaplan (1999) sees the value in recognising a subjective understanding of how individuals may view their behaviour as resilient. This is particularly important when considering how differently understandings of resilient behaviour in ‘troubled’ youth may be perceived across cultures and places (Ungar, 2004). The case studies cited in this paper further underline the importance of accepted versions of resilience should not be ‘tied to the normative judgements relating to particular outcomes’ (Kaplan, 1999, p. 31) This is before we consider the added challenges that Brian—as a young Black man—also had to navigate. Whilst a full analysis of the intersection of racial, gendered and class dynamics lies beyond the scope of the current paper, it is with confidence that we point to the presence of resilience for global‐majority participants in contending with racial inequalities and defencive attitudes from well‐meaning professionals.^7^ But for Brian, Liam, Samantha, and many other of the young people in the PROMISE study, resilience was demonstrated by simply ‘getting on’ with life and dealing with the various challenges without escalating the conflict.

### 1.4 Reframing resilience: Towards safe‐uncertainty

Many of the participants had experienced trauma, victimisation and abuse. Their day‐to‐day lives and longer‐term futures were characterised by uncertainty. Whilst this could be the case for a lot of young people, especially given the uncertainties and difficulties presented by the COVID‐19 pandemic (McKinlay et al., 2022), our participants could be considered particularly vulnerable in many different senses (see Bui & Deakin, 2021) and lacking in the ‘repertoire of traditional resilience resources’ (Castillo, 2003 cited in Siapno, 2009, p. 58) of family and other networks of support. Unsurprisingly, how best to respond to those young people is fraught with disagreement. These disagreements are, in part, due to practice lagging some way behind more progressive theoretical approaches (cf. Marovic & Snyders, 2010) to managing ‘troubled’ youth, which favour negotiation, co‐creation and shared responsibilities.

Some areas within this field remain, at the very least, consciously *coupled* to several assumptions that have underpinned working practices. These include the modernist idea that there exists an ‘absolute truth’, the hierarchical and fixed operation of power, and that knowledge held by those in authority should be respected and followed by those in their charge (Marovic & Snyders, 2010). As such, the supervisory relationships within these areas, specifically involving therapeutic interventions with ‘troubled’ families, have been traditionally informed by approaches that have been firmly rooted in ‘notions of objectivity, understanding by analysis, reductionism, hierarchy, linear causality, dualism, certainty, and absolutism’ (Marovic & Snyders, 2010, p. 36). Under the influence of what has been termed the ‘zeitgeist of postmodernism’, there has been something of a shift in approaches towards practices ‘from first‐order cybernetics, and an objectivist paradigm to a paradigm of second‐order cybernetics and multiple realities, where subjectivity is seen as inevitable, and where the observer perceives, acts, and creates her own reality’ (Marovic & Snyders, 2010, p. 36). In place of judgement or instruction has been a move to negotiation and discussion; hierarchical relationships that prioritise power and knowledge as held by the ‘expert’ or ‘supervisor’ (or similar) are replaced with an appreciation of mutual influence, shared responsibilities, and co‐creation. Objective positions and specific instructions for change make way for negotiations of co‐creation and focus on the context for changes rather than directions regarding specific acts or actions to make changes (Marovic & Snyders, 2010). Hoffman (1993) has suggested that this shift can be characterised by some additional traits, such as replacing the hierarchical approach with one focusing on collaboration, appreciating the wider context of an individual's situation, and moving away from identifying a specific change that needs to occur to thinking more broadly about the context and how this can be managed (see also Hayward, 1996). In short, these long‐held beliefs around objectivity and the existence of a singular ‘solution’ that needs to be achieved have been, at least in part, debunked.

The field of family therapeutic services has not been entirely able to embrace this shift to this postmodern paradigm. Whilst there were frustrations noted around the concept of certainty, Mason (1993) argued that therapeutic practice still lagged some way behind theoretical thought. To this end, Mason's (1993, 2019) framework is in line with a more progressive shift. Put simply, the underpinning position is to cultivate a space whereby an individual can feel secure in dealing with the unknown or the unexplored, where uncertainty can be addressed without the young person feeling paralysed by fear or anxiety. To borrow from Bion's (1962, 1967) work, safe‐uncertainty enables the individual to learn to deal with anxieties and fear. Without it, they are left with a ‘nameless dread’ (Bion, 1967, p. 116). Allowing this space for ‘safe‐uncertainty’ (Mason, 1993) can provide a supportive atmosphere that allows positive changes to be set in motion, in spite of any emotional turbulence that may be present. Mason conceives four key positions on the safe/uncertain‐unsafe/certain axis.

Those in the domain of **unsafe‐certainty** are conceived as very fixed in their views and unable or unwilling to consider alternative perspectives. They are usually ‘convinced of the certainty of their points of view… [have] less curiosity of the other, of the perspectives of other people, and … will often try to convince others of the rightness of their perspectives’ (Mason, 2019, p. 346). However, this position risks the individual experiencing what Berlant (2011) termed ‘cruel optimism’; that the goal(s) to which they strive is unobtainable, perhaps due to structural inequalities that render their endeavours fruitless.

Safe‐certainty sees those wishing to achieve a sense of security in their lives seek to maintain a situation that they are familiar or comfortable with. For some young people in this study, safe‐certainty may entail remaining in their comfort zone, which could be continuing to associate with delinquent peers and engaging in anti‐social or criminal behaviour, perpetuating the ‘at risk’ label that they may already be subject to. It can also be argued that inaction, confrontation, resistance (Bottrell, 2007) or repeated deviant behaviour evident amongst some of the participants in response to conflict are mechanisms to help maintain feelings of safety and certainty, thus building and maintaining a (perhaps fragile) sense of resilience. This may seem nonsensical, particularly when the individual knows the consequences of their actions, and that there is potential support on offer to aid desistance. However, it may be that these behaviours provide a net of certainty for them, providing them with a familiar set of cognitive scripts and a certainty of knowledge of the likely outcomes of deviant behaviour (e.g., the consequences of being caught). It may allow them to remain alongside other similarly marginalised young people with whom they have formed a bond or have an—albeit unorthodox—support network, which can have a resilience‐promoting effect (Wexler et al., 2009). It can also provide a sense of security in avoiding the uncertainty of different paths, such as the ones that involve desistance. This ‘hidden resilience’ (Ungar, 2006, 2011) presents modes of (mal)adaptation that are taken to help survive the challenging situations, and potentially guard their wellbeing (Bottrell, 2009). Taking this into account, it is unsurprising that these young people seek to remain in this position. The obvious problem with this is the unpredictability of life; any number of factors can come into play and disrupt these periods of stasis. The result can plunge the young person back into feelings of insecurity, and compound or reignite stresses and anxieties. It dissuades young people from developing everyday tactics of resilience. It is only through ‘exposure to the full range of social and environmental variation’ (Walker, 2020, p. 12) that forms of resilience can be developed. Safe‐certainty is, as Mason (2019) suggests, only ever a temporary solution.

Unsafe‐uncertainty is a worrying position to occupy. Individuals can feel fatalistic about their future, as though they are unable to influence their lives, which may feel chaotic, overwhelming and debilitating. It can be a lonely and insecure position to be in, whereby those in this situation feel unable to talk to others for fear of being judged or viewed negatively and so it becomes something of a silent burden (McKinney, 2020). According to Mason, unsafe‐uncertainty is:

Where the weight of what has to be dealt with… constrains one’s ability to make meaning of what has happened and is happening. It contributes to constraining what might help in developing narratives that encompass possibilities of hope and resilience.

(Mason, 2019 p. 345)

It is a position of **safe‐uncertainty** that is of particular interest to this paper. Safe‐uncertainty can offer ‘a place where doubt, uncertainty, unhelpful difference, can be safely, if at times uncomfortably, explored as part of developing […] more constructive, safer relationships’ (Mason, 2019, p. 347). It offers space for alternative views to be considered, and for previously unsaid or unsayable thoughts and ideas to be voiced without fear of judgement, ridicule, or censure. To hold the position of safe‐uncertainty is to recognise that circumstances are fluid, subject to change and easily influenced by external sources and internal decisions. It invites deliberate cultivation of uncertainty as a way of building a sense of security, thus providing confidence in taking steps towards positive change, which may present something of the unknown. It is for professionals (e.g., social workers) to take the approach of ‘knowing not to know’, whereby expertise and knowledge are combined with ‘a collaborative stance of uncertainty and respect for the client's knowledge and narrative’ (Larner, 2000, p. 72), allowing for ‘authoritative doubt’, which allows the professional to hold on to both their expertise whilst also acknowledging the uncertainty of some situations, and giving consideration to multiple variables involved and the different potential solutions. Positions of safe‐certainty and unsafe‐certainty both encourage the importance of expertise and certainty in knowing, however, safe‐uncertainty instead embraces the idea of ‘not knowing’ and, borrowing from Cecchin's (1987) use of curiosity, opens the young person and the adult working with them up to the different meanings of actions and events, alternative possibilities, and explanations.

There is, therefore, a need to promote a nuanced version of resilience in order to be inclusive of less obvious indicators, moving beyond more traditional understandings of behaviour that are thought to be indicative of resilience. Resilience among young people may be evident in a range of non‐normative behaviour, inaction, and so on; behaviour that may be repeated despite efforts from various interventions. Resilience requires a more nuanced approach that appreciates and accounts for the complexities involved in surviving adverse situations. This, in turn, embraces the more complex appreciations of resilience, counter to those more psychological, individualistic, and normative understandings of resilience. Resilience, where this equates to overcoming or managing to live with adversity, can be manifested in a multitude of ways, and as such needs to be understood as a multifaceted concept that should be viewed within the wider social context of the young person involved, using an approach that appreciates the diversity of resilient behaviour that includes those which may be less normative.

Mason's work has had a significant impact within the broader field of contemporary therapeutic practices (Vetlesen, 2006), particularly in the areas of systemic and family therapies, where it has encouraged a shift from an ‘expert’‐driven focus on finding definitive solutions to embracing the complexity of human experiences (Cecchin et al., 1994; Dallos & Draper, 2010). This fosters a more flexible, responsive, collaborative and exploratory therapeutic process (Cooper & McLeod, 2011), which is particularly useful in addressing complex ethical issues (Bouverie & Scott, 2001) and family dynamics (Mason, 2005). Mason's model has had some direct influence on the discussion about the broader concept of resilience (cf. Norris et al., 2008; Walsh, 2016), primarily through its contribution to systemic and ecological thinking, although it has not been a central or widely cited framework in these discussions. That said, its principles align with key concepts in resilience studies and chimes with ideas linked to adaptability and the management of uncertainty, which feature in many discussions on resilience (Rutter, 2012; Ungar, 2008, 2011). The safe‐uncertainty model has also inspired the development of other therapeutic models that emphasise the importance of balancing structure with flexibility. For instance, narrative therapy and solution‐focused therapy often incorporate elements of safe‐uncertainty, encouraging clients to explore multiple perspectives and co‐construct new meanings (Vetlesen, 2001).

### 1.5 Working with (Safe)Uncertainty

Having established the potential for the Safe‐Uncertainty model (Mason, 2019), the question remains as to how to promote a more positive and sustainable form of resilience, through the position of safe‐uncertainty, amongst young people labelled as ‘risky’.

Enabling a move to these positions of safe‐uncertainty may provide the individual with the ability to access and utilise available resources that can support resilience building (Ungar, 2011) but there also needs to be the capacity of local governments and other societal agencies to recognise what is needed in terms of this support and resources (Bottrell, 2009). Our research revealed a chaotic pattern of relationships between staff and young people. Alongside these relationships, which ‘often had a confusing maelstrom of both stigmatising and supporting elements involved’, most of the young people ‘experienced multiple and often overlapping sites of conflict, which frequently involved more than one (semi)authority figure’ (Deakin, Fox, & Harragan, 2022, p. 106).

The less successful interventions tended to come from the statutory sector, though not exclusively, and usually demonstrated several traits, including feelings of not being listened to and ignored, perceptions of being (negatively) judged or stigmatised, or let down. Liam frequently and explicitly expressed dissatisfaction with most of the authority figures that he had encountered (‘Some of them are all right, but they don't fucking listen’, Liam, 15, UK). Similarly, Samantha and Princess had also had difficult dealings with authority figures, in particular social workers:

[B]ut I feel like they've, like, split us up and, like, never really cared about us. 'Cause when we've asked them for things, like to help us see the kids more, they've just never really done it. […] I feel like they don't listen.

(Princess, 20, UK)

I hate them [social workers] …. And they judge you as well. […] So, they're gonna judge me, aren't they? 'Cause my mum and dad didn't look after us.

(Samantha, 24, UK)

For those who had experienced hardships and felt failed by the services designed to support them, it was not uncommon to see that they had developed a protective adaptation to inoculate themselves against any further potential hurt and trauma. A sense of safe‐uncertainty was evident in discussions with Brian and Liam. Both described their instinct to trust themselves rather than rely on others:

You got to stick to what you know and what you're doing yourself, innit like. I mean like, I just know that no one's got me as more than I've got myself, innit. […] I’m in my own lane, aren't I?

(Brian, 18, UK)

I’d rather do shit on me own.

(Liam, 15, UK)

The participants' mistrust of these formal systems aligns with Ungar's (2006) discussion of hidden resilience as an adaptive strategy. From this perspective, their decision to disengage from these systems is not necessarily a sign of failure but rather a protective mechanism in response to past negative experiences and systemic failures. This hard shell of resilience that had been (understandably) formed by some of the participants risked leading them to remain in ‘risky’ situations or behave in a manner that threatened (further) ‘trouble’ with criminal justice agencies.

Whilst it is apparent that many of the features of supportive relationships continue to be recognised as exceptionally valuable in encouraging desistance and social engagement (not least by the current study, see Deakin, Fox, & Harragan, 2022; cf. Johns et al., 2017; Case & Haines, 2015; Creaney, 2015; Robinson, 2016; McNeill et al., 2012), the notion of cultivating a safe‐uncertain space for young people to occupy remains under‐explored in the existing youth justice discussions.

Opting to encourage the status of safe‐uncertainty, and in doing so, increasing resilience to current and future difficulties, means moving away from more rigid understandings of what ‘solutions’ will ‘solve’ a particular problem, and instead allows for more fluid and collaborative narratives to emerge whereby new explanations and aims can be formed. It moves away from the risk‐based approaches that view ‘troubled’ youth as ‘restricted bundles of risks’ (Haines & Case, 2015, p. 87), reduced to the problems that they represent (Robinson, 2016). Instead, by moving away from positions of expertise and knowing, and eschewing the persistence towards a narrowly conceived ‘solution’ (Gray, 2013), the wider context of that person's life, including their contribution towards understanding this, becomes part of the focus.

Across the range of interventions, the PROMISE project revealed the impact that positive relationships and interventions can have on young people (Deakin, Fox, & Harragan, 2022), particularly when they had been guided towards positions of safe‐uncertainty. Several features demonstrated this. For example, three of the four case studies saw the participants discuss the places, usually provided by the voluntary sector, that made them feel welcome and at ease. The provision of such ‘safe’ spaces existed both in, and beyond, a physical sense. They offered a place to meet friends, talk to staff, or just be by themselves. They also provided a safe emotional space that allowed respectful explorations of ideas and identities where staff would hear young people's views:

[youth worker] is not going to judge you. She gives you her opinion, but she never puts her opinion above yours. In other words, if you have to make a mistake, she will let you make mistakes. She will warn you that you might be wrong with it, but she won’t scorn your previous idea.

(Paco, 16, Spain)

She [probation officer] was supportive. She talked, advised.

(Aare, 22, Estonia)

The concept of ‘safe’ space is a widely used one across different settings, and even across the sites involved in this research project. Several criticisms of its use and application highlight the complexities surrounding the ‘safe space’ discourse (Flensner & Von der Lippe, 2019). Amongst these is the lack of recognition of the subjectivity of safety; What constitutes a ‘safe space’ is highly subjective. In recognising that the idea of ‘safe spaces’ cannot be used unproblematically, the terms ‘safe(r) spaces’ and ‘safe enough spaces’ are preferred, which convey the sense that these spaces can offer a place to promote safe‐uncertainty but recognise that there is no universal, objective definition of what constitutes a ‘safe space’; what feels ‘safe’ for some may feel exclusionary to others (Ahmed, 2012) and as Butler (2004) has explored, how different bodies experience feelings of safety and vulnerability across spaces is variable, based on social structures of power and privilege. Those, like some of the young people in PROMISE study, who have experienced trauma may have heightened sensitivities to environments that others may perceive as safe, illustrating the subjectivity of safety. Some arguments (Haidt & Lukianoff, 2018; Roth, 2014) advocate against forms of ‘safe’ spaces, suggesting that they conversely promote emotional fragility by sheltering individuals from difficult or uncomfortable discussions. ‘Safe’ spaces, from this perspective, can be seen as an overprotection that impedes people's ability to engage with real‐world challenges or develop resilience. There is, therefore, some tension between protecting individuals from harm and promoting resilience through exposure to challenges, which Mason's model addresses through the notion of ‘safe‐uncertainty’.

There appeared to be three notable factors in the more successful relationships between participants and the intervention staff members: trust, empathy and respect (Deakin, Fox, & Matos, 2022):

Everyone has always judged the way I talk, my gestures… Then when I first got here it was like: wow, these people are not paying attention to the way I talk but to what I am saying!

(Marc, 21, Estonia)

These interventions were frequently compared to being like ‘our family’ (focus group, UK) or offering a home‐like sanctuary where participants felt ‘affection’ (Telma, 19, Portugal) and ‘safe and secure’ (focus group, UK). This provided something akin to ‘shock absorbers’ that offered ‘critical safety nets’ (Moser, 1998, p. 11).

The result of these actions that enabled a move to safe‐uncertainty was that the young people concerned demonstrated their resilience through feelings of acceptance and a certain amount of confidence in their future actions:

It's made me more confident with people. Like I speak to people now, like, yeah. It's made me a bit more open‐minded and stuff. Like, as in like meeting new people and that.

(Sophie, 23, UK)

It is not sufficient to simply acknowledge that resilience may take unorthodox, anti‐social forms. There needs to be a move away from the more risk‐based approaches and towards measures that recognise safe‐uncertainty (Mason, 1993) amongst those deemed to be ‘at risk’. Encouraging a position of safe‐uncertainty enables young people to develop healthy adaptations to ‘survive unhealthy circumstances’ (Bottrell, 2009, p. 325), even when those adaptations do not fit the neoliberal ideals of resilient behaviour. This appreciation of a more complex concept of resilience is compatible with Mason's model, in that it values how uncertain, changeable, and ambiguous the future may be for these young people.

## 2 CONCLUSION

The young people in our study—like many—have faced a plethora of challenges in a rapidly changing world. Economic uncertainties, stories of parental indifference/neglect, addiction and/or abuse, and educational struggles alongside coming into the focus of criminal justice agencies have resulted in a hostile environment that the young people in this study needed to navigate. Despite differences (and similarities) across and within each of the four case studies that this paper has utilised, the narratives elicited point to various reactive mechanisms or adaptations utilised by the young people to enable the often‐significant difficulties and conflicts to be managed. By doing this, they are demonstrating resilience, albeit in various, sometimes less conventional forms, whether that is manifested as rebelling, opting to remain in vulnerable positions of conflict, ‘latent rejection’ (Deakin, Fox, & Harragan, 2022; Deakin, Fox, & Matos, 2022), ‘doing nothing’ (Corrigan, 1993), ‘hidden resilience’ (Ungar, 2004, 2006, 2011) or through the more commonly recognised resilience discourse of overcoming adversity or doing better than expected despite the presence of challenging situations (Ungar, 2004).

Resilience is cast as an essential attribute of neoliberal life, particularly within the context of the cost‐of‐living crisis and austerity that has beset recent years (Gill & Orgad, 2018). But questions abound as to what form resilience may take, who defines what counts as resilient behaviours and what purpose does this serve. The promotion of resilience as a desirable individual trait in neoliberal societies raises important broader questions about the redistribution of responsibility from the state to individuals. By emphasising personal resilience, there is a risk of normalising adversity and hardship as inevitable aspects of life that individuals must simply learn to overcome. The emphasis on resilience in policy and practice also raises questions about the ultimate beneficiaries of such approaches. While ostensibly aimed at supporting those deemed vulnerable, the focus on building personal resilience can potentially shift attention away from addressing the root causes of social problems. This serves to maintain the status quo by encouraging adaptation to difficult circumstances rather than challenging or changing the systems that produce those circumstances.

Furthermore, the dominant narratives around resilience often privilege certain forms of ‘bouncing back' or overcoming adversity that align with neoliberal values of self‐reliance, productivity, and economic success. This narrow definition fails to recognise or validate the diverse ways in which young people demonstrate resilience in their daily lives, particularly those from the PROMISE study employed strategies that may appear counterintuitive or problematic. In this vein, and in the context of austerity and reduced social services, the promotion of resilience can be seen as a cost‐effective alternative to more comprehensive support systems. This raises ethical concerns about the adequacy of expecting individuals to develop resilience ‐ in its more conventional form ‐ in the face of significant structural barriers and limited resources.

A more nuanced understanding of resilience that acknowledges its diverse manifestations and the complex interplay between individual agency and structural factors is needed. This expanded conceptualisation should inform interventions that not only support individual coping mechanisms but also address the broader social and economic conditions that necessitate such resilience in the first place. This resilience reframing and the use of Mason's work has wide‐ranging implications, from youth support to public health, disaster preparedness, and international development. Future research could examine how cultural differences might influence expressions of resilience and the applicability of the safe‐uncertainty model in diverse global contexts.

This article argues that not only do the participants of our study demonstrate a capacity for resilience, despite it not necessarily conforming to the confines of more normative social constructions that feature in policy and practice. The reactions to conflict, ‘marginalisation’ and victimisation or harmful behaviour, such as parental neglect, discussed within this paper were far from homogenous or in keeping with heroic characterisations of resilience. A nuanced appreciation of resilience is critical when developing interventions that work with ‘at‐risk’ young people. Such understandings will impact the development of resources and interventions (Bottrell, 2009).

When resilience, in this more complex configuration, is not invested in—be that in an emotional or more practical sense—it can mitigate against a young person developing better self‐esteem and confidence, which can, in turn, bolster their ability to make better choices and foster life skills. Mason's (1993) model can offer a framework for interventions and organisations that work with ‘troubled’ young people. Rather than seeing uncertainty as something to be avoided at all costs, it is critical for young people's development, particularly those who are considered ‘risky’ and/or who may be wrestling with multiple disadvantages and difficulties, that the investment in ‘the certain’ be disrupted. This allows a better preparation for the future; the difficulties, disruptions, and disasters that most people experience, which may be felt more keenly or with greater frequency by those coming from disadvantaged backgrounds. The concept of safe‐uncertainty, central for fostering resilience, contrasts sharply with the risk‐averse practices common in criminal justice, health, social care, and governance systems. These systems prioritise control, standardisation, and liability management, limiting the flexibility needed to navigate life's uncertainties. Safe‐uncertainty, as highlighted in this paper, balances creating a safe(r) space for exploration while acknowledging uncertainty as natural and unavoidable in everyday life. Through imposing rigid approaches to risk management, the ability for professionals to promote this within their work with young people is constrained and stifled. This leaves little room for nuanced decision‐making. Consequently, the current institutional environment poses a significant barrier to fostering resilience‐building by reducing opportunities for individuals to productively engage with uncertainty.

It is, therefore, naïve to suggest that encouraging an approach of safe‐uncertainty is appropriate or even achievable for all young people or those who work with them. There is a multiplicity of factors, alongside the predominance of risk rhetoric, that may prohibit such an approach, particularly within the wider political context that is preoccupied with ‘tough talk’ rhetoric, and with the backdrop of ever‐threatening economic cutbacks. This worrying combination places pressure on both statutory and third‐sector providers in each of the four countries which, in a time so utterly consumed and shaped by difficulties associated with the cost of living crisis and the remnants of the COVID‐19 pandemic, presents a significant challenge for professionals to navigate.

## Supporting information

Table S1

## Data Availability

The data that support the findings of this study are available on request from the corresponding author. The data are not publicly available due to privacy or ethical restrictions.

## ENDNOTES

^1^
Although there remain some anomalies as to how aspects of the concept are theorised, operationalised and applied (Weaver, 2019), the field of desistance has been a fruitful and rapidly grown area of research over the past 2 decades in particular. Desistance is now largely thought to be a gradual and dynamic process, perhaps better explained as ‘the long‐term abstinence from crime among individuals who had previously engaged in a persistent pattern of criminal offending’ (Maruna, 2001, p. 26). Desistance can be influenced by various social, psychological, and structural factors, such as age, social bonds (family, work), and personal identity changes (Laub & Sampson, 2003; Maruna, 2001).

^2^
These have included a wide variety of factors, including levels of intelligence, temperament, problem‐solving abilities, motivations, and other personality traits (cf. Benard, 1995 cited in Brooks, 2006; Leffert et al., 1998; Rutter, 1987).

^3^
A detailed consideration of the latter can offer a more comprehensive understanding of how resilience is shaped by social power dynamics (cf. Cabrera Martinez, et al., 2022; Clark, 2022; Fleming & Ledogar, 2008; Mohaupt, 2009).

^4^
The Promoting Youth Involvement and Social Engagement (PROMISE) project was a multinational study that focused on the promoters and inhibitors of young people's social engagement, in various forms, incorporating 12 organisations across 10 European countries. The qualitative element of this mixed‐method research involved taking a meta‐ethnographic approach (Pilkington, 2018).

^5^
Further explanation of the nature of the group as well as the research findings for each individual case can be found at: https://www.promise.manchester.ac.uk/en/home‐page/.

^6^
See Table 1 for details of the case studies, participants and data collection. More detailed information on the demographics and contexts of the case studies is discussed by Deakin, Fox, and Matos (2022).

^7^
See DiAngelo's (2011) concept of ‘White Fragility’ for an example of these behaviours.

^8^
Wider contextual information regarding each country participating in the project can be found in the National Context Reports PROMOTING YOUTH INVOLVEMENT AND SOCIAL ENGAGEMENT (PROMISE) Reports from the four countries included in this analysis begin on pages 6, 48, 59 and 102. Statistical information on each country can be found in the Short Country Reports PROMOTING YOUTH INVOLVEMENT AND SOCIAL ENGAGEMENT (PROMISE). These provide an overview of the general ‘state of the country's health’; the situation that young people face; how young people feel about their situation; and what, if anything, they are doing to change it. Reports from the four countries included in this analysis begin on pages 18, 89, 110 and 170.

## References

[bjos13164-bib-0001] Aburn, G. , Gott, M. , & Hoare, K. (2016). What is resilience? An integrative review of the empirical literature. Journal of Advanced Nursing, 72(5), 980–1000. 10.1111/jan.12888 26748456

[bjos13164-bib-0002] Ahmed, S. (2012). On being included: Racism and diversity in institutional life. North Carolina. Duke University Press.

[bjos13164-bib-0003] Allen, K. , & Bull, A. (2018). Following policy: A network ethnography of the UK character education policy community. Sociological Research Online, 23(2), 152–172. 10.1177/1360780418769678

[bjos13164-bib-0004] Andres, L. , & Round, J. (2015). The role of ‘persistent resilience’ within everyday life and polity: Households coping with marginality within the ‘big society’. Environment and Planning A: Economy and Space, 47(3), 676–690. 10.1068/a46299

[bjos13164-bib-0005] Bateman, T. (2020). The state of youth justice 2020: An overview of trends and developments. National Association for Youth Justice.

[bjos13164-bib-0006] Berlant, L. (2011). Cruel optimism. North Carolina. Duke University Press.

[bjos13164-bib-0007] Bion, W. (1962). Learning from experience. Karnac.

[bjos13164-bib-0008] Bion, W. (1967). Second thoughts: Selected papers on psycho‐analysis. Penguin Books.

[bjos13164-bib-0009] Bottrell, D. (2007). Resistance, resilience and social identities: Reframing “problem youth” and the problem of schooling. Journal of Youth Studies, 10(5), 597–616. 10.1080/13676260701602662

[bjos13164-bib-0010] Bottrell, D. (2009). Understanding ‘marginal’ perspectives towards a social theory of resilience. Qualitative Social Work, 8(3), 321–339. 10.1177/1473325009337840

[bjos13164-bib-0011] Bouverie, P. J. , & Scott, S. (2001). Practising safe uncertainty: Addressing ethical dilemmas in family therapy. Australian and New Zealand Journal of Family Therapy, 22(3), 133–140.

[bjos13164-bib-0012] Boyden, J. , & Cooper, E. (2007). Questioning the power of resilience: Are children up to the task of disrupting the transmission of poverty? Chronic Poverty Research Centre (CPRC) Working Paper 73.

[bjos13164-bib-0013] Bradbury‐Jones, C. , Breckenridge, J. , Clark, M. , Oliver, H. , Wagstaff, C. , & Taylor, J. (2017). The state of qualitative research in health and social science literature: A focused mapping review and synthesis. International Journal of Social Research Methodology, 20(6), 627–645. 10.1080/13645579.2016.1270583

[bjos13164-bib-0014] Brooks, J. E. (2006). Strengthening resilience in children and youths: Maximizing opportunities through the schools. Children and Schools, 28(2), 69–76. 10.1093/cs/28.2.69

[bjos13164-bib-0015] Bui, L. , & Deakin, J. (2021). What we talk about when we talk about vulnerability and youth crime: A narrative review. Aggression and Violent Behavior, 58, 101605. Article 101605. 10.1016/j.avb.2021.101605

[bjos13164-bib-0016] Bull, A. , & Allen, K. (2018). Introduction: Sociological interrogations of the turn to character. Sociological Research Online, 23(2), 392–398. 10.1177/1360780418769672

[bjos13164-bib-0017] Burman, E. (2018). (Re)sourcing the character and resilience manifesto: Suppressions and slippages of (re)presentation and selective affectivities. Sociological Research Online, 23(2), 416–437. 10.1177/1360780418769671

[bjos13164-bib-0018] Butler, J. (2004). Precarious life: The powers of mourning and violence. Verso Books.

[bjos13164-bib-0019] Cabrera Martinez, L. , Barrita, A. , & Wong‐Padoongpatt, G. (2022). A systematic literature review on the resilience reported by BIPOC in the face of discrimination. Spectra Undergraduate Research Journal, 2(1), 1–12. 10.9741/2766-7227.1012

[bjos13164-bib-0020] Case, S. , & Haines, K. (2015). Children first, offenders second positive promotion: Reframing the prevention debate. Youth Justice, 15(3), 226–239. 10.1177/1473225414563154

[bjos13164-bib-0021] Cecchin, G. (1987). Hypothesising, circularity and neutrality revisited: An invitation to curiosity. Family Process, 26(4), 405–413. 10.1111/j.1545-5300.1987.00405.x 3319683

[bjos13164-bib-0022] Cecchin, G. , Lane, G. , & Ray, W. A. (1994). The cybernetics of prejudices in the practice of psychotherapy. Karnac Books.

[bjos13164-bib-0023] Clark, J. (2022). Resilience in the context of conflict‐related sexual violence and beyond: A “sentient ecology” framework. British Journal of Sociology, 73(2), 352–369. 10.1111/1468-4446.12931 35218670 PMC9305244

[bjos13164-bib-0024] Cooper, M. , & McLeod, J. (2011). Pluralistic counselling and psychotherapy. SAGE Publications.

[bjos13164-bib-0025] Corrigan, P. (1993). Doing nothing. In S. Hall & T. Jefferson (Eds.), Resistance through rituals: Youth subcultures in post‐war britain. Routledge.

[bjos13164-bib-0026] Creaney, S. (2015). Still working with ‘involuntary clients’ in youth justice. British Journal of Community Justice, 13(1), 41–54.

[bjos13164-bib-0027] Crenshaw, K. (1989). Demarginalizing the intersection of race and sex: A Black feminist critique of antidiscrimination doctrine, feminist theory and antiracist politics. University of Chicago Legal Forum, 1(8), 139–167.

[bjos13164-bib-0028] Dallos, R. , & Draper, R. (2010). An introduction to family therapy: Systemic theory and practice (3rd ed.). McGraw‐Hill Education.

[bjos13164-bib-0029] Davoudi, S. (2012). Resilience: A bridging concept or a dead end? Planning Theory and Practice, 13, 299–307.

[bjos13164-bib-0030] Deakin, J. , Fox, C. , & Harragan, A. (2022). Help or hindrance? Rethinking interventions with ‘troubled youth’. International Journal of Law in Context, 18(1), 100–115. 10.1017/s1744552322000064

[bjos13164-bib-0031] Deakin, J. , Fox, C. , & Matos, R. (2022). Labelled as ‘risky’ in an era of control: How young people experience and respond to the stigma of criminalized identities. European Journal of Criminology, 19(4), 653–673. 10.1177/1477370820916728

[bjos13164-bib-0032] DeVerteuil, G. , & Golubchikov, O. (2016). Can resilience be redeemed? Resilience as a metaphor for change, not against change. City, 20(1), 143–151. 10.1080/13604813.2015.1125714

[bjos13164-bib-0033] DiAngelo, R. (2011). White fragility. International Journal of Critical Pedagogy, 3(3), 54–70.

[bjos13164-bib-0034] Estêvão, P. , Calado, A. , & Capucha, L. (2017). Resilience: Moving from a "heroic" notion to a sociological concept. Sociologia ‐ Problemas e Praticas, 85, 9–25.

[bjos13164-bib-0035] Evans, B. , & Reid, J. (2013). Dangerously exposed: The life and death of the resilient subject. Resilience, 1(2), 83–98. 10.1080/21693293.2013.770703

[bjos13164-bib-0036] Fergus, S. , & Zimmerman, M. A. (2005). Adolescent resilience: A framework for understanding healthy development in the face of risk. Annual Review of Public Health, 26(1), 399–419. 10.1146/annurev.publhealth.26.021304.144357 15760295

[bjos13164-bib-0037] Fitzpatrick, C. (2011). What is the difference between “desistance” and “resilience”? Exploring the relationship between two key concepts. Youth Justice, 11(3), 221–234. 10.1177/1473225411420528

[bjos13164-bib-0038] Fleming, J. , & Ledogar, R. (2008). Resilience, an evolving concept: A review of literature relevant to aboriginal research. Pimatisiwin, 6(2), 7–23. 20963184 PMC2956753

[bjos13164-bib-0039] Flensner, K. K. , & Von der Lippe, M. (2019). Being safe from what and safe for whom? A critical discussion of the conceptual metaphor of ‘safe space’. Intercultural Education, 30(3), 275–288. 10.1080/14675986.2019.1540102

[bjos13164-bib-0040] Fox, C. , Deakin, J. , Spencer, J. , & Acik, N. (2022). Encountering authority and avoiding trouble: Young migrant men’s narratives of negotiation in Europe. European Journal of Criminology, 19(4), 791–810. 10.1177/1477370820924627

[bjos13164-bib-0041] Gill, R. , & Orgad, S. (2018). The amazing bounce‐backable woman: Resilience and the psychological turn in neoliberalism. Sociological Research Online, 23(2), 477–495. 10.1177/1360780418769673

[bjos13164-bib-0042] Gilligan, R. (2000). Adversity, resilience and young people: The protective value of positive school and spare time experiences. Children and Society, 14(1), 37–47. 10.1002/(sici)1099-0860(200002)14:1<37::aid-chi564>3.3.co;2-n

[bjos13164-bib-0043] Gray, P. (2013). Assemblages of penal governance, social justice and youth justice partnerships. Theoretical Criminology, 17(4), 517–534. 10.1177/1362480613496450

[bjos13164-bib-0044] Green, S. , Calverley, A. , & O’Leary, N. (2021). A new approach for researching victims: The “strength‐growth‐resilience” framework. British Journal of Criminology, 61(3), 852–871. 10.1093/bjc/azaa093

[bjos13164-bib-0045] Haidt, J. , & Lukianoff, G. (2018). The coddling of the American mind: How good intentions and bad ideas are setting up a generation for failure. Penguin Press.

[bjos13164-bib-0046] Haines, K. , & Case, S. (2015). Positive youth justice: Children first, offenders second. Policy Press.

[bjos13164-bib-0047] Harris, L. , Chu, E. , & Ziervogel, G. (2017). Negotiated resilience. Resilience, 6(3), 196–214. 10.1080/21693293.2017.1353196

[bjos13164-bib-0048] Hayward, M. (1996). Is second order practice possible? Journal of Family Therapy, 18(3), 219–242. 10.1111/j.1467-6427.1996.tb00046.x

[bjos13164-bib-0049] Hoffman, L. (1993). Exchanging voices. Karnac books.

[bjos13164-bib-0050] Hutcheon, E. , & Lashewicz, B. (2014). Theorizing resilience: Critiquing and unbounding a marginalizing concept. Disability & Society, 29(9), 1383–1397. 10.1080/09687599.2014.934954

[bjos13164-bib-0051] Hutcheon, E. , & Lashewicz, B. (2015). Are individuals with disabilities and their families “resilient”? Deconstructing and recasting a well‐intended concept. Journal of Social Work in Disability & Rehabilitation, 14(1), 41–60. 10.1080/1536710x.2015.989560 25569019

[bjos13164-bib-0052] Johns, D. F. , Williams, K. , & Haines, K. (2017). Ecological youth justice: Understanding the social ecology of young people’s prolific offending. Youth Justice, 17(1), 3–21. 10.1177/1473225416665611

[bjos13164-bib-0053] Joseph, J. (2013). Resilience as embedded neoliberalism: A governmentality approach. Resilience, 1(1), 38–52. 10.1080/21693293.2013.765741

[bjos13164-bib-0054] Kaplan, H. B. (1999) ‘Toward an understanding of resilience: A critical review of definitions and models’ in In M. D. Glantz and J. L. Johnson (eds.) Resilience and development: Positive life adaptations. : Kluwer Academic/Plenum.

[bjos13164-bib-0055] Larner, G. (2000). Towards a common ground in psychoanalysis and family therapy: On knowing not to know. Journal of Family Therapy, 22(1), 61–82. 10.1111/1467-6427.00138

[bjos13164-bib-0056] Laub, J. H. , & Sampson, R. J. (2003). Shared beginnings, divergent lives. Harvard University Press.

[bjos13164-bib-0057] Leffert, N. , Benson, P. L. , Scales, P. C. , Sharma, A. R. , Drake, D. R. , & Blyth, D. A. (1998). Developmental assets: Measurement and prediction of risk behaviors among adolescents. Applied Developmental Science, 2(4), 209–230. 10.1207/s1532480xads0204_4

[bjos13164-bib-0058] Lenkens, M. , Rodenburg, G. , Schenk, L. , Nagelhout, G. E. , Van Lenthe, F. J. , Engbersen, G. , Sentse, M. , Severiens, S. , & Van De Mheen, D. (2020). “I need to do this on my own” resilience and self‐reliance in urban at‐risk youths. Deviant Behavior, 41(10), 1330–1345. 10.1080/01639625.2019.1614140

[bjos13164-bib-0059] Lenkens, M. , van Lenthe, F. J. , Schenk, L. , Magnée, T. , Sentse, M. , Sabine, M. , Severiens, S. , Engbersen, G. , & Nagelhout, G. E. (2019). Experiential peer support and its effects on desistance from delinquent behavior: Protocol paper for a systematic realist literature review. Systematic Reviews, 8(1), 119–133. 10.1186/s13643-019-1036-2 31103043 PMC6525969

[bjos13164-bib-0060] Luthar, S. S. , Cicchetti, D. , & Becker, B. (2000). The construct of resilience: A critical evaluation and guidelines for future work. Child Development, 71(3), 543–562. 10.1111/1467-8624.00164 10953923 PMC1885202

[bjos13164-bib-0061] Marovic, S. , & Snyders, F. (2010). Cybernetics of supervision: A developmental perspective. The Clinical Supervisor, 29(1), 35–50. 10.1080/07325221003730277

[bjos13164-bib-0062] Maruna, S. (2001). Making good: How ex‐convicts reform and rebuild their lives. American Psychological Association Books.

[bjos13164-bib-0063] Mason, B. (1993). Towards positions of safe uncertainty. Human Systems: The Journal of Systemic Consultation Practices and Management, 4, 198–200.

[bjos13164-bib-0064] Mason, B. (2005). The safe‐uncertainty house: From modernist to postmodernist approaches in systemic practice. Journal of Family Therapy, 27(2), 158–167.

[bjos13164-bib-0065] Mason, B. (2019). Re‐Visiting safe uncertainty: Six perspectives for clinical practice and the assessment of risk. The Association for Family Therapy and Systemic Practice Journal of Family Therapy, 41(3), 343–356. 10.1111/1467-6427.12258

[bjos13164-bib-0066] Masten, A. S. (1994). Resilience in individual development: Successful adaptation despite risk and adversity: Challenges and prospects. In M. Wang & E. Gordon (Eds.), Educational resilience in inner city America: Challenges and prospects (pp. 3–25). Lawrence Erlbaum.

[bjos13164-bib-0067] McKeown, A. , Hai Bui, D. , & Glenn, J. (2021). A social theory of resilience: The governance of vulnerability in crisis‐era neoliberalism. European Journal of Cultural and Political Sociology, 9(1), 112–132. 10.1080/23254823.2021.1997616

[bjos13164-bib-0068] McKinlay, A. R. , May, T. , Dawes, J. , Fancourt, D. , & Burton, A. (2022). You’re just there, alone in your room with your thoughts’: A qualitative study about the psychosocial impact of the COVID‐19 pandemic among young people living in the UK. BMJ Open, 12(2), e053676. 10.1136/bmjopen-2021-053676 PMC8829834 35140155

[bjos13164-bib-0069] McKinney, J. (2020). Towards safe(R) uncertainty: An offering for managers and supervisors during a pandemic. Murmurations: Journal of Transformative Systemic Practice, 3(1), 113–129. 10.28963/3.1.17

[bjos13164-bib-0070] McNeill, F. , Farrall, S. , Lightowler, C. , & Maruna, S. (2012). How and why people stop off ending: Discovering desistance. Institute for Research and Innovation in the Social Sciences. Available at: http://www.iriss.org.uk/sites/default/files/iriss‐insight‐15.pdf

[bjos13164-bib-0071] Mohaupt, S. (2009). Review article: Resilience and social exclusion. Social Policy and Society, 8(1), 63–71. 10.1017/s1474746408004594

[bjos13164-bib-0072] Moser, C. O. (1998). The asset vulnerability framework: Reassessing urban poverty reduction strategies. World Development, 26(1), 1–19. 10.1016/s0305-750x(97)10015-8

[bjos13164-bib-0073] Mu, G. (2020). Sociologising resilience through bourdieu's field analysis: Misconceptualisation, conceptualisation, and reconceptualisation. British Journal of Sociology of Education, 42(1), 15–31. 10.1080/01425692.2020.1847634

[bjos13164-bib-0074] Mu, G. (2021). Time to ring the death knell for agency and resilience? Some sociological rethinkings of inclusive education. International Journal of Disability, Development and Education, 68(6), 822–830. 10.1080/1034912x.2020.1866751

[bjos13164-bib-0075] Mythen, G. (2012). Identities in the third space? British Journal of Sociology, 63(3), 393–411. 10.1111/j.1468-4446.2012.01416.x 22950460

[bjos13164-bib-0076] Norris, F. H. , Stevens, S. P. , Pfefferbaum, B. , Wyche, K. F. , & Pfefferbaum, R. L. (2008). Community resilience as a metaphor, theory, set of capacities, and strategy for disaster readiness. American Journal of Community Psychology, 41(1–2), 127–150. 10.1007/s10464-007-9156-6 18157631

[bjos13164-bib-0077] Pelling, M. (2010). Adaptation to climate change: From resilience to transformation. Routledge.

[bjos13164-bib-0078] Pilkington, H. (2018). Employing meta‐ethnography in the analysis of qualitative data sets on youth activism: A new tool for transnational research projects? Qualitative Research, 18(1), 108–130. 10.1177/1468794117707805

[bjos13164-bib-0079] Robinson, A. (2016). The resilience motif: Implications for youth justice. Youth Justice, 16(1), 18–33. 10.1177/1473225415587601

[bjos13164-bib-0080] Roth, M. S. (2014). Beyond the university: Why liberal education matters. Yale University Press.

[bjos13164-bib-0081] Rutter, M. (1987). Psychosocial resilience and protective mechanisms. American Journal of Orthopsychiatry, 57(3), 316–331. 10.1111/j.1939-0025.1987.tb03541.x 3303954

[bjos13164-bib-0082] Rutter, M. (2012). Resilience: Causal pathways and social ecology. In M. Ungar (Ed.), The social ecology of resilience: A handbook of theory and practice. Springer.

[bjos13164-bib-0083] Schoon, I. (2006). Risk and resilience; adaptations in changing times. Cambridge University Press.

[bjos13164-bib-0084] Siapno, J. A. (2009). Living through terror everyday resilience in east timor and aceh. Social Identities, 15(1), 43–64. 10.1080/13504630802692903

[bjos13164-bib-0085] Ungar, M. (2004). A constructionist discourse on resilience: Multiple contexts, multiple realities among at risk children and youth. Youth & Society, 35(3), 341–360. 10.1177/0044118x03257030

[bjos13164-bib-0086] Ungar, M. (2006). Final report: International resilience project. Available online at http://www.resilienceproject.org

[bjos13164-bib-0087] Ungar, M. (2008). Resilience across cultures. British Journal of Social Work, 38(2), 218–235. 10.1093/bjsw/bcl343

[bjos13164-bib-0088] Ungar, M. (2011). The social ecology of resilience: Addressing contextual and cultural ambiguity of a nascent construct. American Journal of Orthopsychiatry, 81(1), 1–17. 10.1111/j.1939-0025.2010.01067.x 21219271

[bjos13164-bib-0089] Ungar, M. (2013). The impact of youth‐adult relationships on resilience. International Journal of Child, Youth and Family Studies, 4(3), 328. 10.18357/ijcyfs43201312431

[bjos13164-bib-0090] Vetlesen, A. J. (2001). Perception, empathy, and judgment: An inquiry into the preconditions of moral performance. Penn State University Press.

[bjos13164-bib-0091] Vetlesen, A. J. (2006). Public concern and the problem of violence: Theoretical reflections. Journal of Scandinavian Studies in Criminology and Crime Prevention, 7(2), 153–167.

[bjos13164-bib-0092] Walker, B. (2020). Resilience: What it is and not. Ecology and Society, 25(2), 1–3.32523609

[bjos13164-bib-0093] Walklate, S. , Mythen, G. , & McGarry, R. (2012). States of resilience and the resilient state. Current Issues in Criminal Justice, 24(2), 185–204. 10.1080/10345329.2012.12035954

[bjos13164-bib-0094] Walsh, F. (2002). Bouncing forward: Resilience in the aftermath of september 11. Family Process, 41(1), 34–36. 10.1111/j.1545-5300.2002.40102000034.x 11924088

[bjos13164-bib-0095] Walsh, F. (2016). Strengthening family resilience (3rd ed.). Guilford Press.

[bjos13164-bib-0096] Weaver, B. (2016). Offending and desistance: The importance of social relations. Routledge.

[bjos13164-bib-0097] Weaver, B. (2019). Understanding desistance: A critical review of theories of desistance. Psychology, Crime and Law, 25(6), 641–658. 10.1080/1068316x.2018.1560444

[bjos13164-bib-0098] Wexler, L. M. , DiFluvio, G. , & Burke, T. K. (2009). Resilience and marginalized youth making a case for personal and collective meaning making as part of resilience research in public health. Social Science & Medicine, 69(5), 65–70. 10.1016/j.socscimed.2009.06.022 19596503

[bjos13164-bib-0099] White, I. , & O’Hare, P. (2014). From rhetoric to reality: Which resilience, why resilience, and whose resilience in spatial planning? Environment and Planning C: Government and Policy, 32(5), 934–950. 10.1068/c12117

[bjos13164-bib-0100] Zolkoski, S. M. , & Bullock, L. M. (2012). Resilience in children and youth: A review. Children and Youth Services Review, 34(12), 2295–2303. 10.1016/j.childyouth.2012.08.009

