# Supplementary material for: Breaking good? Young people's mechanisms of resilience, resistance and control
Source: Br J Sociol. 2024 Nov 21;76(2):316–35. doi: 10.1111/1468-4446.13164 (PMC11890428; doi:10.1111/1468-4446.13164)
Supplement: Supplementary file 1 — Table S1 [file BJOS-76-316-s001.docx]

**Revisions**

| **Comment** | **Response** |
| --- | --- |
| **Editor:** While the paper as written makes a persuasive intervention into the literature on resilience, it doesn't yet articulate its contribution to sociology more broadly as clearly as it should, particularly in the introduction and also in the abstract. Bearing in mind that this is a general interest sociological journal, I'd ask you to revise the paper so that it is of potential interest to non-specialist audiences. There are a number of ways to do this.  The first is in your articulation of the stakes of the research.  The second is in your theoretical framing/intervention -- here you make it clear that resilience is ubiquitous, which makes it potentially broadly important, but you could do more to indicate how your theoretical/conceptual development can travel beyond this study/area.  The third is in a more expansive discussion of the implications, e.g. elaborating after this point: "Resilience is cast as an essential attribute of neoliberal life, particularly within the context of the cost-of-living crisis and austerity that has beset recent years (Gil and Orgad, 2018). But questions abound as to what form resilience may take, who defines what counts as resilient behaviours and what purpose does this serve."  Ideally you would strive to achieve all three. | Thank you for this comment and particularly for giving some direction as to where/how these improvements could be made. It was very helpful.  To address the issues raised, we have rewritten and added to the discussions in several places throughout the paper:  Abstract: The abstract has been rewritten accordingly, and the following sentence added to further underline the broader applicability and interest of the paper: *This approach towards a more sustainable resilience could be valuable in other fields dealing with those populations labelled as ‘vulnerable’, ‘problematic’ or ‘disadvantaged’, and it can, we argue, enhance decision-making skills, and promote the development of robust support networks.*  Pages 2-3: The last paragraph on page 2 and the first paragraph on page 3 have been added to highlight the importance of the paper’s argument and relevance beyond the immediate context of the study.  Page 5: This page has been mostly rewritten to incorporate more discussion on the motivations and implications for political usage of ‘resilience’.  Conclusion, pages 20-22: The conclusion has been rewritten and extended to allow for elaboration of the implications of resilience within the context of neoliberalism (e.g. tied in with the quote from Gil and Orgad, 2018). It also suggests how this discussion may be relevant to other fields of study/practice. |
| **Reviewer 1:** please explain the term desistance, which is used as if it is known and clear. I understand this comes from the criminal justice (and perhaps youth justice) system but this is unlikely to be obvious to all readers | Apologies for not including this in the original submission. There is a brief description of ‘desistance’ on p.1-2, which is accompanied by a short footnote. The supporting references have all been added to the bibliography. |
| It might be worth emphasising that the position of safe-uncertainty is precisely what is NOT espoused by all our health, social care and other governance systems, which operate with a kind of risk averse defensive practice that makes such provision tricky. I appreciate this is what the author(s) are perhaps saying but it could be clearer! | Thank you for this observation. We have added a few lines on pages 6-7 to emphasize the risk-averse nature of these systems. We then return to it in more detail make this point clearer and have also returned to it in more detail towards the end of the conclusion on page 21, where we highlight that the safe-uncertainty approach contrasts significantly by that embedded within these various systems. |
| I would have appreciated some further context on and for Mason's domain(s) of practice and some comments on how influential (or not) they are in resilience debates. Their link with family systems and therapy may be relevant to comment upon, for example? | Following this helpful suggestion, much of page 12 and a paragraph on page 15 (immediately above the subheading) is now devoted to this and outlines both the place of Mason’s work within his own domain, and the influence it has (or hasn’t!) had both within and beyond that. |
| The reference to Bion was interesting and the whole question of 'not knowing', or as is often discussed in therapeutic and educational contexts, sitting with the discomfort. It might have been useful to comment on how this imports a  psychoanalytic register. Crossing such disciplinary and paradigmatic boundaries is in itself perhaps an instance of navigating safe(ish) uncertainties? | We are very grateful for this comment. It is very much in line with some discussions that Author 1 has had with another colleague on the topic of discomfort and how some of this paper, and the wider study from which is draws upon, relates to a more psychoanalytical perspective. This has prompted a return to some psychoanalytic works, starting with Bion’s importance of ‘not knowing’ (and also, perhaps, more accurately ‘sort of knowing’) and moving on to Winnicott’s work on holding environments and transitional space*,* and also Lacan’s focus on the unconscious and the Real (the unassimilated and often traumatic parts of experience). As a result, there is scope for another, different paper that focuses more readily on this aspect of the project and the work on sitting with discomfort, which we would like to develop. It would be challenging to fully do this comment from the reviewer justice within the current paper (and the word limit), although we would very much like to develop this line of thought and discussion in much greater depth. |
| the question of safe spaces is always tricky - and might be better qualitied as 'relatively safe'? | This is a very good point, thank you, which we very much agree with. We have added a paragraph to the ‘safe space’ discussion on page 18, and opt for ‘safe enough’ or ‘safe(r) spaces’ instead of using ‘safe spaces’ unproblematically. |
| **Reviewer 2:** Engagement with Existing Literature: The author notes previous studies on what has been termed “hidden” resilience. I encourage the author to delve deeper into how their findings conceptually relate to these earlier studies. Currently, the paper acknowledges the existence of these studies without fully engaging with the conceptual connections to the present findings. | We agree that there is a lot more to explore on ‘hidden’ resilience in relation to our study. We have drawn out some additional points from the analysis that align more clearly with Ungar’s work in particular – see pages 9 and 17 – which is in addition to the other discussions on ‘hidden’ resilience, and the related points. Within the confines of the current paper, it is challenging to devote more space to this point, although it may be something that warrants further discussion in a later publication. Thank you for giving us food for thought with this comment |
| Clarification of "Gold Standard" of Resilience: In the introduction and literature review, the author mentions a “gold standard” of resilience. However, a detailed explanation of what exactly this gold standard entails and why it is problematic only appears on page 5. Given its central importance, this explanation should be moved to an earlier part of the paper for better contextual understanding. | Thank you for this observation. We have moved this part of the discussion towards the beginning of the introduction (pgs 1-2), which provides a much better contextualisation from the outset. |
| Separation of "Unsafe-Certain" Concept: The concept of “unsafe-certain” should be placed in its own paragraph to enhance readability and emphasize its significance. | Good point - the ‘unsafe-certainty’ section has been moved so it is a paragraph on its own (see page 13). We have also placed each of the four concepts within Mason’s model in bold type when mentioning for the first time within this section. |
